# Intercellular adhesion molecule-1 enhances the therapeutic effects of MSCs in a dextran sulfate sodium-induced colitis models by promoting MSCs homing to murine colons and spleens

**DOI:** 10.1186/s13287-019-1384-9

**Published:** 2019-08-23

**Authors:** Xin Li, Qian Wang, Li Ding, Yu-Xing Wang, Zhi-Dong Zhao, Ning Mao, Chu-Tse Wu, Hua Wang, Heng Zhu, Shou-Bin Ning

**Affiliations:** 1 Air Force Medical Center, PLA, Road Fucheng 30, Beijing, 100142 People’s Republic of China; 2 People’s Liberation Army General Hospital, Road Fuxing 28, Beijing, 100853 People’s Republic of China; 3 Beijing Institute of Basic Medical Sciences, Road Taiping 27, Beijing, 100850 People’s Republic of China; 4 Beijing Institute of Radiation Medicine, Road Taiping 27, Beijing, 100850 People’s Republic of China; 5 Jizhong Energy Xingtai MIG General Hospital, Road Bayi 202, Xingtai, 054000 People’s Republic of China

**Keywords:** Inflammatory bowel disease, Mesenchymal stem cells, Intercellular cell adhesion molecule-1, Homing

## Abstract

**Background:**

To investigate the therapeutic effect of intercellular adhesion molecule (ICAM)-1-modified mesenchymal stem cells (MSCs) in a mouse model of inflammatory bowel disease (IBD) induced by dextran sulfate sodium.

**Methods:**

Primary MSCs and ICAM-1-overexpressing MSCs (C3 cells) were generated in vitro. The IBD mouse model was induced with drinking water containing dextran sulfate sodium for 7 days. For stem cell therapy, mice were randomly assigned to six experimental groups: the control group, IBD group, primary MSC group, C3 group, C3-vector group, and C3-ICAM-1 group. Mice were given a single injection of 1 × 10^6^ primary MSCs or gene-modified MSCs via the tail vein on day 3 of DDS administration. The general conditions of the mice in each group were observed. Additionally, the pathological changes in the colon were observed and scored. Primary MSCs and gene-modified MSCs were stained with the fluorescent dye CM-DIL before injection into the tail vein of mice. The distribution of infused cells in IBD mice was observed in frozen sections. Mechanistically, the polarization of Th1, Th2, Th17, and regulatory T cells (Tregs) in the spleen was determined by flow cytometry. Moreover, the mRNA expression levels of IBD-related immune factors in splenocytes were measured by quantitative PCR.

**Results:**

A single injection of MSCs promoted general recovery and reduced pathological damage in IBD mice. Additionally, ICAM-1-overexpressing MSCs had stronger therapeutic effects than ICAM-1^low^ MSCs. Furthermore, the in vivo distribution analysis results indicated that a higher number of ICAM-1-overexpressing MSCs homed to the colon and spleen of IBD mice. Moreover, the delivery of ICAM-1 overexpressing MSCs decreased the numbers of Th1 and Th17 cells but increased the number of Tregs in the spleen of IBD mice. The quantitative PCR analysis results revealed that an infusion of ICAM-1-overexpressing MSCs influenced the expression of spleen-derived immune factors by remarkably reducing the mRNA levels of IFN-γ and IL-17A and increasing the mRNA level of Foxp3.

**Conclusions:**

Our results demonstrate that ICAM-1-modified mesenchymal stem cells (MSCs) remarkably alleviate inflammatory damage in IBD mice by promoting MSC homing to the target and immune organs. The findings suggest that ICAM-1 is required to maintain the therapeutic effects of MSCs in IBD treatment and identified a novel role of ICAM-1 in inflammatory diseases.

**Electronic supplementary material:**

The online version of this article (10.1186/s13287-019-1384-9) contains supplementary material, which is available to authorized users.

## Introduction

Inflammatory bowel disease (IBD) is an intractable autoimmune disease that leads to abdominal pain, diarrhea, fever, or other symptoms that may be caused by chronic inflammation of the digestive system [1, 2]. Currently, the conventional medications include salicylic acid, corticosteroids, immunosuppressive agents, and antibiotics [3, 4]. These therapies may offer temporary remission but their curative effects are not obvious and adverse reactions, such as psoriasis, drug-induced cytotoxicity, and hypersensitivity, may arise in response to the treatment [3, 4].

Mesenchymal stem cells (MSCs) are prototypical adult stem cells that can self-renew and differentiate into multiple types of cells and tissues in vitro and in vivo. In recent years, MSCs have been shown to have powerful immunomodulatory effects and to stimulate tissue repair capacity; MSCs have been suggested as a promising tool for treatment of IBD [5, 6]. Additionally, MSC homing to the target organ is important for the therapeutic effects in vivo [7, 8]. Intercellular adhesion molecule (ICAM)-1 has been demonstrated to play the crucial roles in the specific and efficient immune responses [9]. ICAM-1 belongs to the immunoglobulin superfamily of cell adhesion molecules (CAMs) that guide the homing of various immune cells to the proper anatomical location within secondary lymphoid organs. Physiologically, ICAM-1 is expressed at a low level in MSCs [9, 10]. However, ICAM-1 expression in MSCs sharply increases in inflammatory microenvironments. Additionally, ICAM-1 upregulation has been demonstrated to contribute to the immunosuppressive effects of MSCs [9, 10]. In our previous work, we constructed ICAM-1-overexpressing MSCs using the mouse MSC line C3H10T1/2 (C3 cells). We found that elevated ICAM-1 expression remarkably suppressed osteogenic differentiation of MSCs [11]. Additionally, ICAM-1 enhanced MSC migration in the transwell system in vitro and promoted MSC migration to the injured thyroid gland in vivo [12].

Due to the inflammatory characteristics of IBD and the therapeutic effects of MSCs in inflammatory diseases, we hypothesized that ICAM-1 in MSCs may contribute to IBD treatment. Therefore, in the present study, we treated IBD mice with primary MSCs and ICAM-1-overexpressing MSCs. The in vivo distribution of MSCs, their therapeutic effects, and the underlying mechanisms were investigated in an IBD mouse model.

## Materials and methods

### Animals

BALB/c (H-2Kd) mice (male, 1–2 weeks of age) used for the cultivation of primary MSCs were purchased from the Animal Center of the Academy of Military Medical Sciences (Beijing, China). BALB/c (H-2Kd) mice (male, 6–8 weeks of age) with a body weight of 20 g were purchased from Beijing Vital River Laboratory Animal Technology Co., Ltd. The mice were raised in a second-class animal room of the Advanced Laboratory Animal Center of the Academy of Military Medical Sciences and fed with sterilized feed and drinking water. All animals received care according to the Guide for the Care and Use of Laboratory Animals. The protocol was approved by the Committee on the Ethics of Animal Experiments of the Academy of Military Medical Sciences.

### Cell preparation

Primary MSCs were isolated from mouse compact bone and expanded according to a published protocol [13]. MSCs at passages 3–6 were used for the experiments unless indicated otherwise. The immunophenotypes and multipotency of the isolated MSCs were detected according to our previous protocol [13]. Briefly, the immunophenotypes of isolated MSCs were analyzed by flow cytometry. Phycoerythrin (PE)-conjugated monoclonal antibodies against mouse CD29, CD31, CD44, CD86, CD105, CD140a (PDGF receptor a), and MHC-Ia (major histocompatibility complex [MHC] class II) and allophycocyanin-conjugated antibodies against mouse stem cell antigen-1 (Sca-1; all products were purchased from eBio-Science) were used. To detect multiple differentiation of MSCs, the osteogenic, adipogenic, and chondrogenic differentiation assays were performed. Alkaline phosphatase staining and Von Kossa staining were used to determine osteogenic activity; Oil Red O staining was used to determine adipogenic differentiation, and toluidine blue staining was performed to determine chondrogenic activity.

The mouse MSC cell line (C3 cells, from C3H mice) was purchased from the American Type Culture Collection (Manassas, VA, USA). ICAM-1-overexpressing mouse MSCs were prepared according to a previously described protocol [11].

### MSC treatment of IBD mice

Mice were randomly assigned to six experimental groups including a control group, IBD group, primary MSC group, C3 group, C3-vector group, and C3-ICAM-1 group (*n >* 6 in each group). Mice in the control group were watered with deionized water. The rest of the mice were given a 5% DSS solution (Sigma-Aldrich, St. Louis, MO, USA) for 7 days to generate the IBD experimental mouse model, and then, their water source was changed to deionized water [14, 15]. Fresh DSS solution was replaced every other day. On the third day of the treatment, the mice in the primary MSC group, C3 group, C3-vector group, and C3-ICAM-1 group were injected with 1 × 10^6^ cells in 500 μl of PBS via the tail vein. The mice in the control group and IBD group were injected with the same volume of PBS.

### General observation and histopathological evaluation of IBD mice

The general status (stool traits, mental state, and activity) of mice in each group were observed. The weight of mice was measured, and the mortality rates were recorded. To perform histopathological evaluation, mice in each group were sacrificed at day 7 post-infusions; the colon was harvested and the length of the colon was measured. Then, the colon was fixed in 10% neutral formaldehyde, embedded in paraffin, sectioned, and stained with hematoxylin and eosin (HE staining). Scoring was performed according to the Scheiffele criteria [15, 16]. To detect the changes in inflammatory cytokine tumor necrosis factor α (TNF-α) in the colon of IBD mice, immunohistochemical staining was performed and the positive areas were calculated by ImageJ.

### MSC distribution in vivo

The primary MSCs, C3 cells, C3-vector cells, and C3-ICAM-1 cells were labeled with fluorescent CM-DIL according to the manufacturer’s protocol. The cells (1 × 10^6^ cells in 500 μl of PBS) were injected into the lateral tail vein of IBD mice. The colon and spleen of the mice were obtained at 24 h and 48 h, respectively. The samples were pruned to the appropriate size, fixed, embedded, frozen, and sectioned. The infused cells are labeled by CM-DIL and are shown in red. All cell nuclei are stained by DAPI and are shown in blue. The sections were observed under a fluorescent microscope, and the average number of red fluorescent cells per three images in triplicate mice was counted at × 200 magnification.

### Flow cytometry

The cell surface expression of ICAM-1 in MSC cell lines was analyzed by flow cytometry with phycoerythrin (PE)-conjugated monoclonal antibodies against mouse ICAM-1.

For intercellular cytokine staining, mouse splenocytes were harvested and cultured with 500 ng/ml ionomycin, 50 ng/ml phorbol 12-myristate 13-acetate (PMA), and 3 mg/ml brefeldin A (Sigma Aldrich) for 6 h. The splenocytes were washed and stained for CD4 and intracellular interferon-γ (IFN-γ), interleukin (IL)-4, or IL-17A. For analysis of regulatory T cells (Tregs), an anti-mouse intranuclear forkhead box P3 (FoxP3) kit (eBioscience, San Diego, CA) was used according to the manufacturer’s protocol. Signals were recorded by flow cytometry with a FACScalibur system (Becton Dickinson), and data were analyzed with the WinMDI 2.9 software.

### Quantitative polymerase chain reaction (PCR) analysis

To assess the effect of MSCs on immune cytokine expression in splenocytes in vivo, nucleated mouse splenocytes were collected 3 days after cell infusions. Total RNA was extracted with TRIzol reagent (Invitrogen) and reverse transcribed using an mRNA-selective PCR kit (TaKaRa). Mouse IFN-γ, IL-4, IL-17A, and Foxp3 cDNA were amplified by real-time PCR using a SYBR Green PCR kit (Sigma). The primer sequences used for the real-time PCR are shown in Additional file 3: Table S1.

### Statistical analysis

Data are presented as the mean values with standard deviations. Statistical significance was analyzed using one-way ANOVA. *P* values less than 0.05 were considered to be significant.

## Results

### The infusion of ICAM-1-overexpressing MSCs dramatically improved the general condition of IBD mice

In this study, the expression of ICAM-1 in C3 cells was determined before cell transplantation. Flow cytometry revealed that more that 90% of the cells were ICAM-1-positive in the ICAM-1-overexpressing MSC group (Additional file 1: Figure S1).

**Fig. 1:**
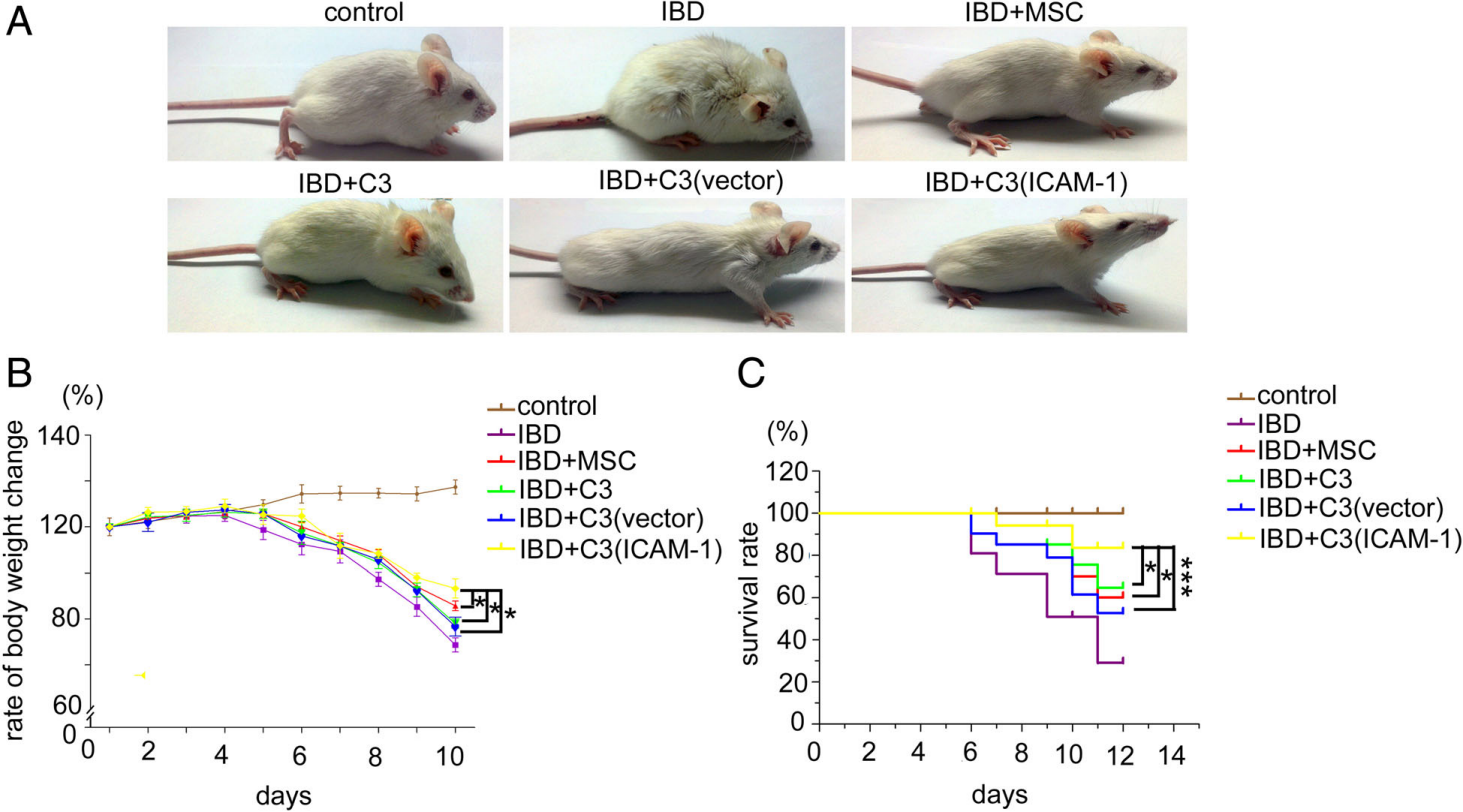
The therapeutic effects of ICAM-1-overexpressing MSCs on the general condition of IBD mice. The depression and food intake (**a**), weight loss (**b**), and the survival rate (**c**) of IBD mice were improved by infusion of primary MSCs, C3 cells, C3-vector cells, and C3-ICAM-1 cells. ICAM-1-overexpressing MSCs have significantly stronger effects than that of primary MSCs, C3 cells, and C3-vector cells (**a**-**c**) (*, *P* < 0.05, ***, *P* < 0.001)

The general condition of mice including their mood, weight, and survival rates were observed every day. Compared to mice in the IBD groups, the IBD mice that received primary MSCs, C3 cells, C3-vector cells, or C3-ICAM-1 cells showed less depression, better food intake, and higher activity (Fig. 1a). Additionally, the weight loss of IBD mice was controlled by MSC infusions (Fig. 1b). Furthermore, MSCs enhanced the survival of IBD mice (Fig. 1c). It should be noted that ICAM-1-overexpressing MSCs had significantly stronger effects than that of primary MSCs, C3 cells, and C3-vector cells (Fig. 1a–c).

### Transplantation of ICAM-1-overexpressing MSCs remarkably alleviated the pathological damage in colon of IBD mice

**Fig. 2:**
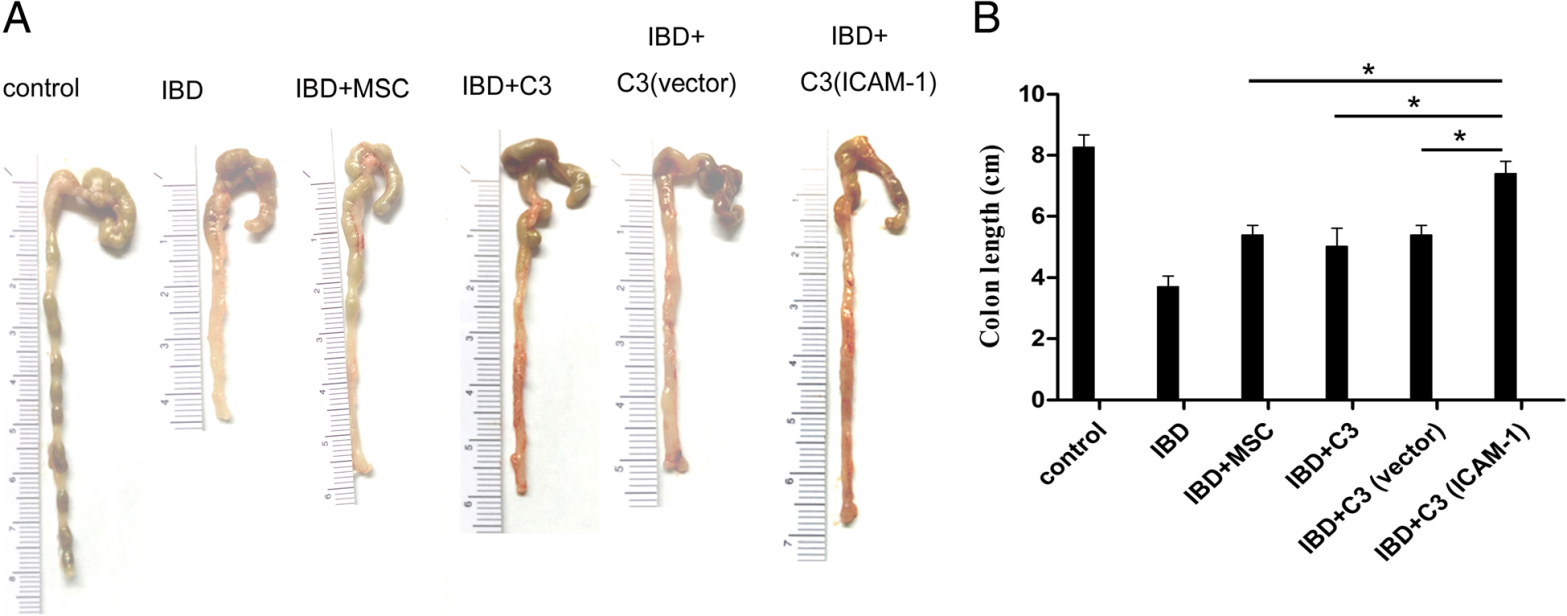
Infusion of ICAM-1-overexpressing MSCs ameliorated contraction and shortening of the colon in IBD mice. Contraction and shortening of the colon in IBD mice was visibly alleviated by infusion of primary MSCs, C3 cells, C3-vector cells, and C3-ICAM-1 cells. ICAM-1-overexpressing MSCs have significantly stronger effects versus the control group than that of primary MSCs, C3 cells, and C3-vector cells (**a** and **b**) (*, *P* < 0.05)

To evaluate the effects of various MSCs on the colon of IBD mice, the colon was examined and its length was measured. As shown in Fig. 2a, the colons of IBD mice were visibly contracted and shortened compared to that in the control group. The colon lesions of IBD mice were remarkably alleviated after MSC infusions. Additionally, colon contraction and shortening were significantly improved in IBD mice treated with ICAM-1-overexpressing MSCs (Fig. 2a, b).

**Fig. 3:**
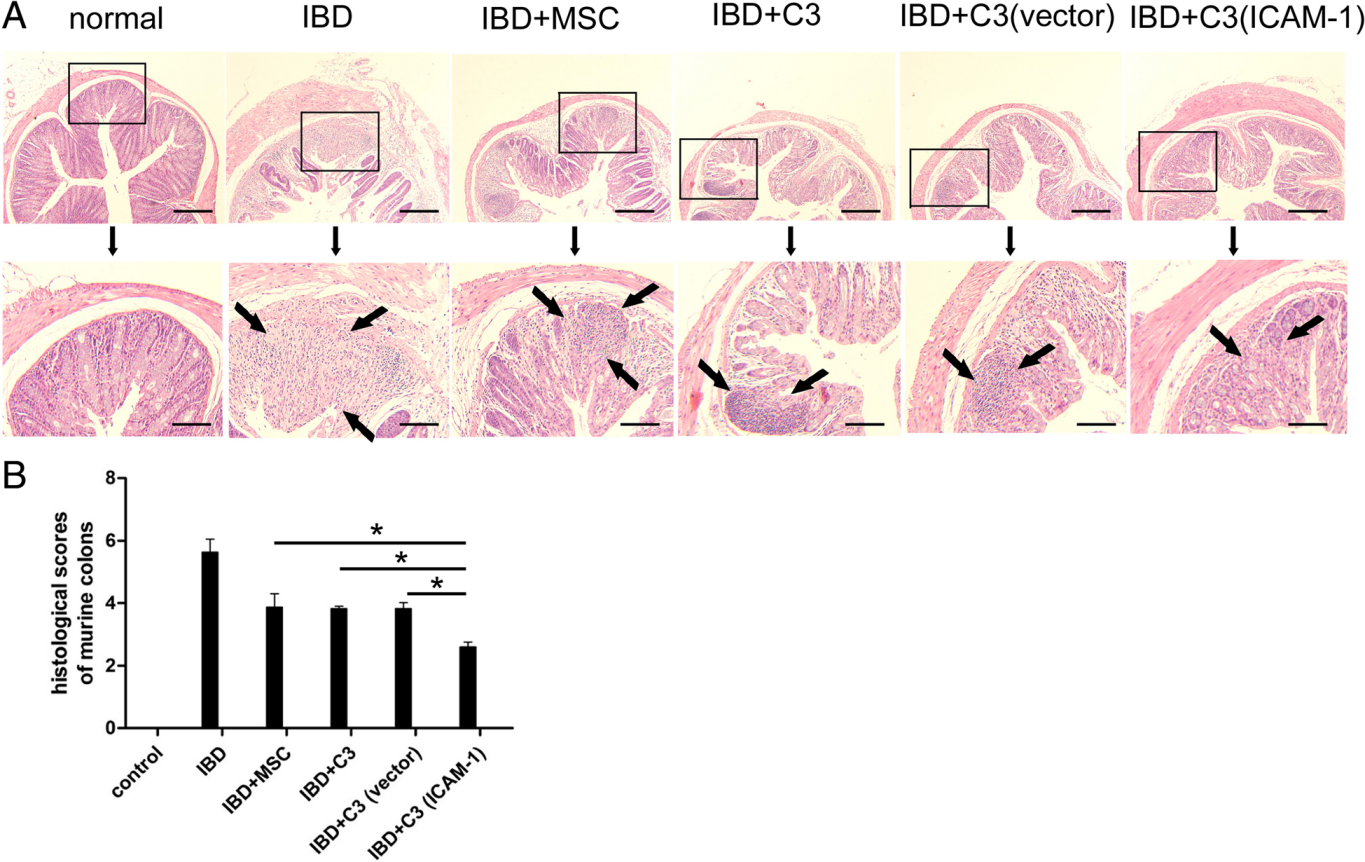
Transplantation of ICAM-1-overexpressing MSCs remarkably alleviated the pathological damage in the colon of IBD mice. The colon structure was almost intact and only a few lymphocytes are detected in the lamina propria in healthy animals; however, severe inflammatory cell infiltration was observed in the villi and lamina propria in the colon of IBD mice. Conversely, MSC infusion reduced the pathological damage in IBD mice and only mild edema of lamina propria and submucosa and a small number of inflammatory cell infiltrations was observed in the colon (**a**). The inflammatory cells in the pictures are shown by arrows. Additionally, the results of pathological scores (Scheiffele criteria) showed that infusion of ICAM-1-overexpressing MSCs remarkably ameliorates the tissue damage (**b**). Bars in **a** represent 500 μm. (*, *P* < 0.05)

To further determine the effects of ICAM-1-overexpressing MSCs on IBD mice, mouse colon was histopathologically examined (Additional file 2). In mice of the control group, the colon structure was intact, and only a few lymphocytes were detected in the lamina propria. However, severe inflammatory cell infiltration was observed in the villi and lamina propria of the colon of IBD mice. It should be noted that edema of the lamina propria and submucosa was the main pathological change in the MSC-treated mice and was accompanied by a minor infiltration of inflammatory cells (Fig. 3a). The pathological scores were assigned according to the Scheiffele criteria. Tissue damage was significantly ameliorated by ICAM-1-overexpressing MSCs (Fig. 3b).

### ICAM-1-overexpressing MSCs have enhanced targeted migration to inflamed colon and spleen in vivo

**Fig. 4:**
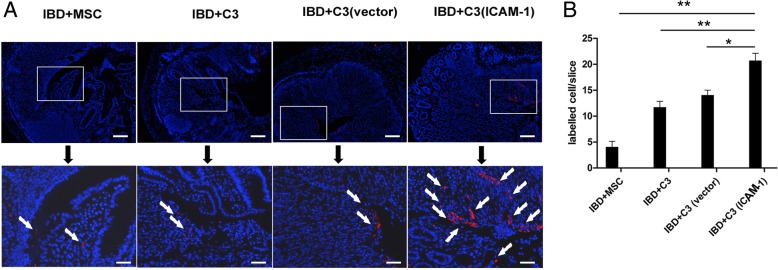
ICAM-1-overexpressing MSCs have enhanced migration to inflamed colon. As shown in **a** and **b**, after intravenous infusion, a higher number of CM-DIL-labeled ICAM-1-overexpressing MSCs is detected in IBD colon in frozen tissue sections. The CM-DIL-labeled ICAM-1 cells are indicated by arrows in **a**. Bars in **a** represent 200 μm. (*, *P* < 0.05, **, *P* < 0.01)

**Fig. 5:**
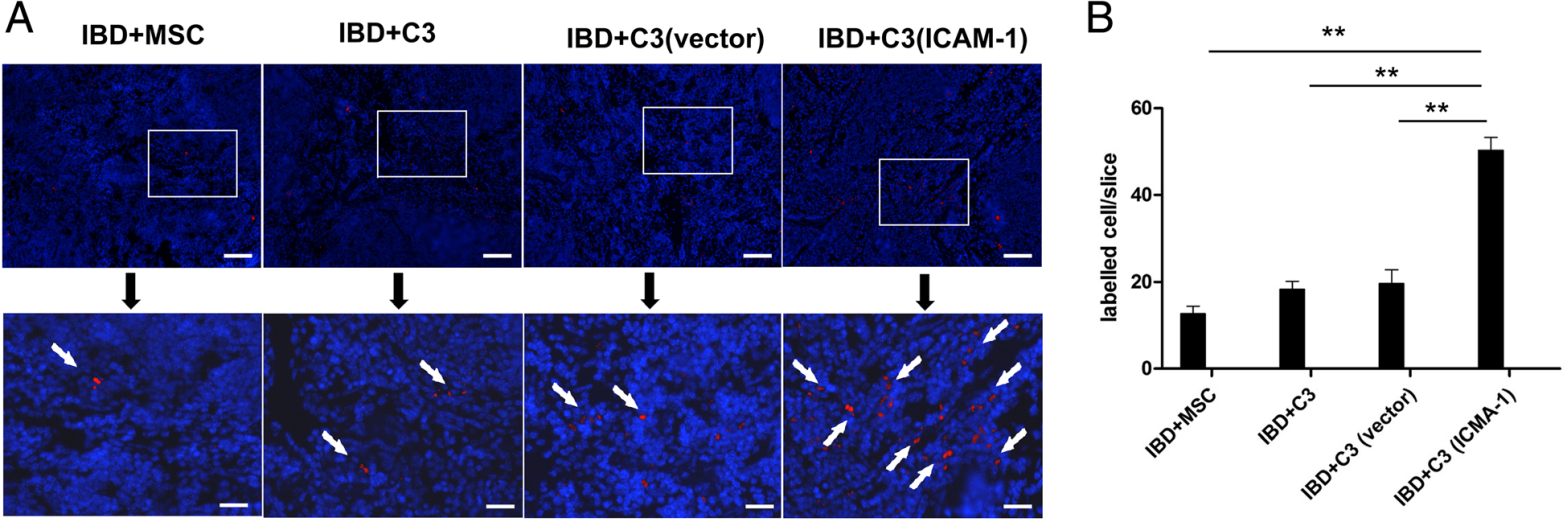
ICAM-1-overexpressing MSCs have significantly increased migration toward the spleen in IBD mice. The infused primary MSCs, C3 cells, C3-vector cells, and C3-ICAM-1 cells are detected in the spleen of IBD mice; however, the results in frozen sections indicate that ICAM-1-overexpressing MSCs showed stronger migration toward the spleen in IBD mice. **a**, **b** ICAM-1-overexpressing MSCs are indicated by arrows in **a**. Bars in **a** represent 200 μm. (*, *P* < 0.05, **, *P* < 0.01)

It is well known that the colon is the main target organ of IBD. To further investigate the mechanisms of MSC-mediated therapeutic effects, the distribution of MSCs in mouse colon was determined using CM-DIL labeling and frozen tissue sectioning. Red fluorescent cells were detected in the colon of IBD mice 24 h after cell infusions. Higher number of MSCs was observed at 48 h in the inflamed colon (Fig. 4 and Fig. 5a). Additionally, compared to the mice in the C3-vector group, MSC localization in the colon was markedly increased in the C3-ICAM-1 group (Fig. 5b). This result indicates that overexpression of ICAM-1 promotes the homing of MSCs to the colon.

**Fig. 6:**
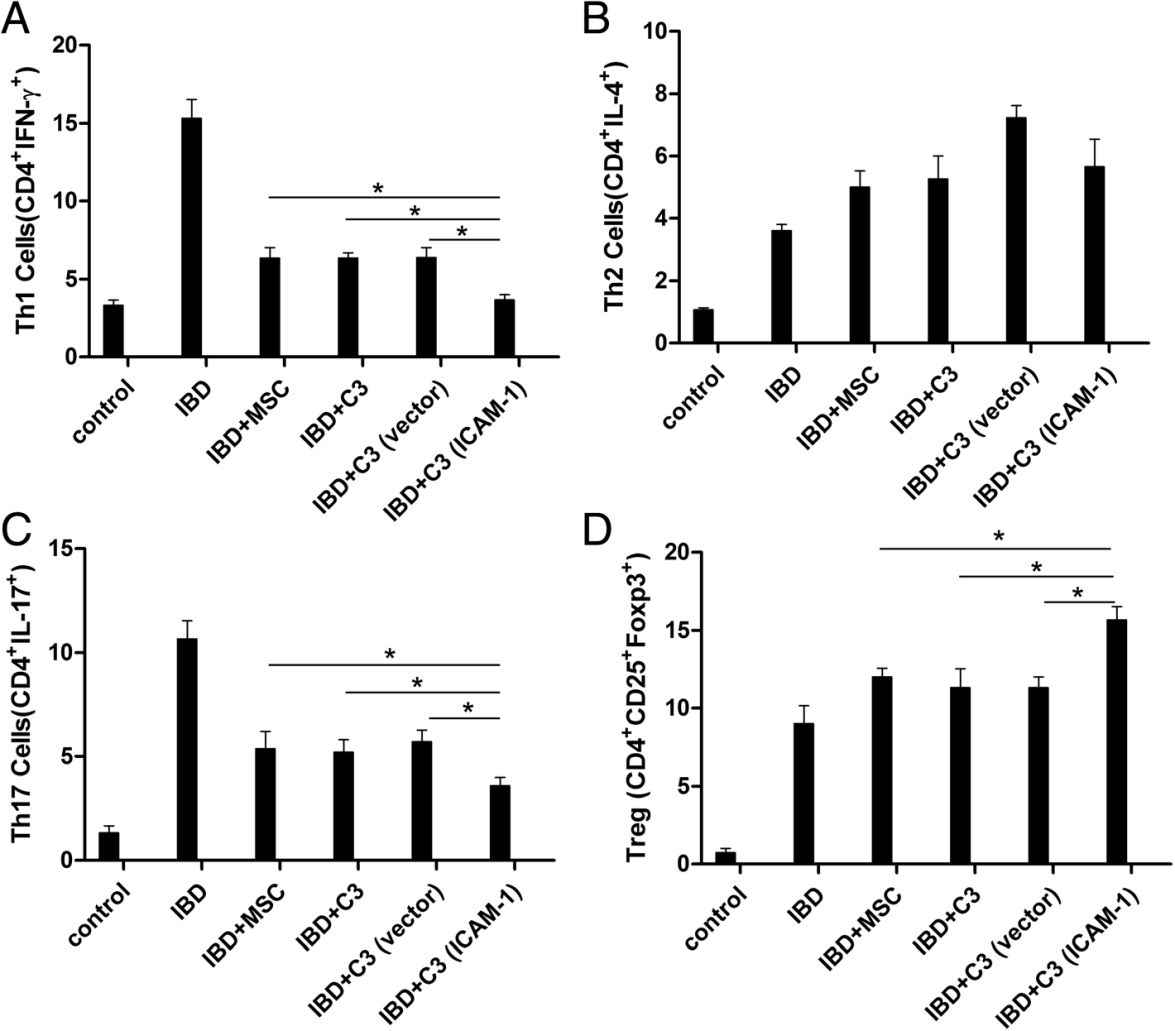
**a**, **b**, **c**, **d** ICAM-1-overexpressing MSCs influence the T cell subpopulations in the spleens of IBD mice. The infusion of primary MSCs, C3 cells, C3-vector cells, and C3-ICAM-1 cells downregulates the percentage of Th1 and Th17 cells and upregulates the percentage of Tregs in the spleen in IBD mice. Additionally, ICAM-1-overexpressing MSCs have stronger modulatory effects on repolarization of T cells. No significant changes in Th2 cells were observed in the current study (Fig. 6) (*, *P* < 0.05)

Accumulating evidence has demonstrated that MSCs migrate to immune organs, control inflammation, and contribute to tissue regeneration through potent immunomodulatory effects on immune cells [17, 18]. Thus, we hypothesized that genetic overexpression of ICAM-1 promotes MSC homing to immune organs, such as the spleen, thereby improving therapeutic effects of MSCs in the mouse model of IBD. The results of frozen sectioning indicated that migration of C3-ICAM-1 cells toward the spleen of IBD mice was significantly increased compared to that of the C3-vector cells (Fig. 6a, b). This increase may contribute to the healing effect of MSCs on the damaged intestine.

### ICAM-1-overexpressing MSCs regulated T cell subpopulations in the spleen and attenuated the release of splenocyte-derived inflammatory cytokines in vivo

Recent studies reported that the polarization of T cell subpopulations is closely involved in the pathological damage in IBD [19, 20]. Considering MSC localization in the spleen and the pivotal role of T cells in IBD, the changes in Th1 (CD4^+^INF-γ^+^ T cells), Th2 (CD4^+^IL-4^+^ T cells), Th17 (CD4^+^IL-17A^+^ T cells), and Treg (CD4^+^CD25^+^Foxp3^+^ cells) cells in the spleen were determined. As shown in Fig. 6, MSC transfusion decreased the percentage of Th1 and Th17 cells but increased the percentage of Tregs in the spleen of IBD mice. Additionally, the modulatory effects of ICAM-1-overexpressing MSCs on the repolarization of T cells were enhanced (Fig. 6). However, no significant changes in Th2 cells were observed in the current study.

**Fig. 7:**
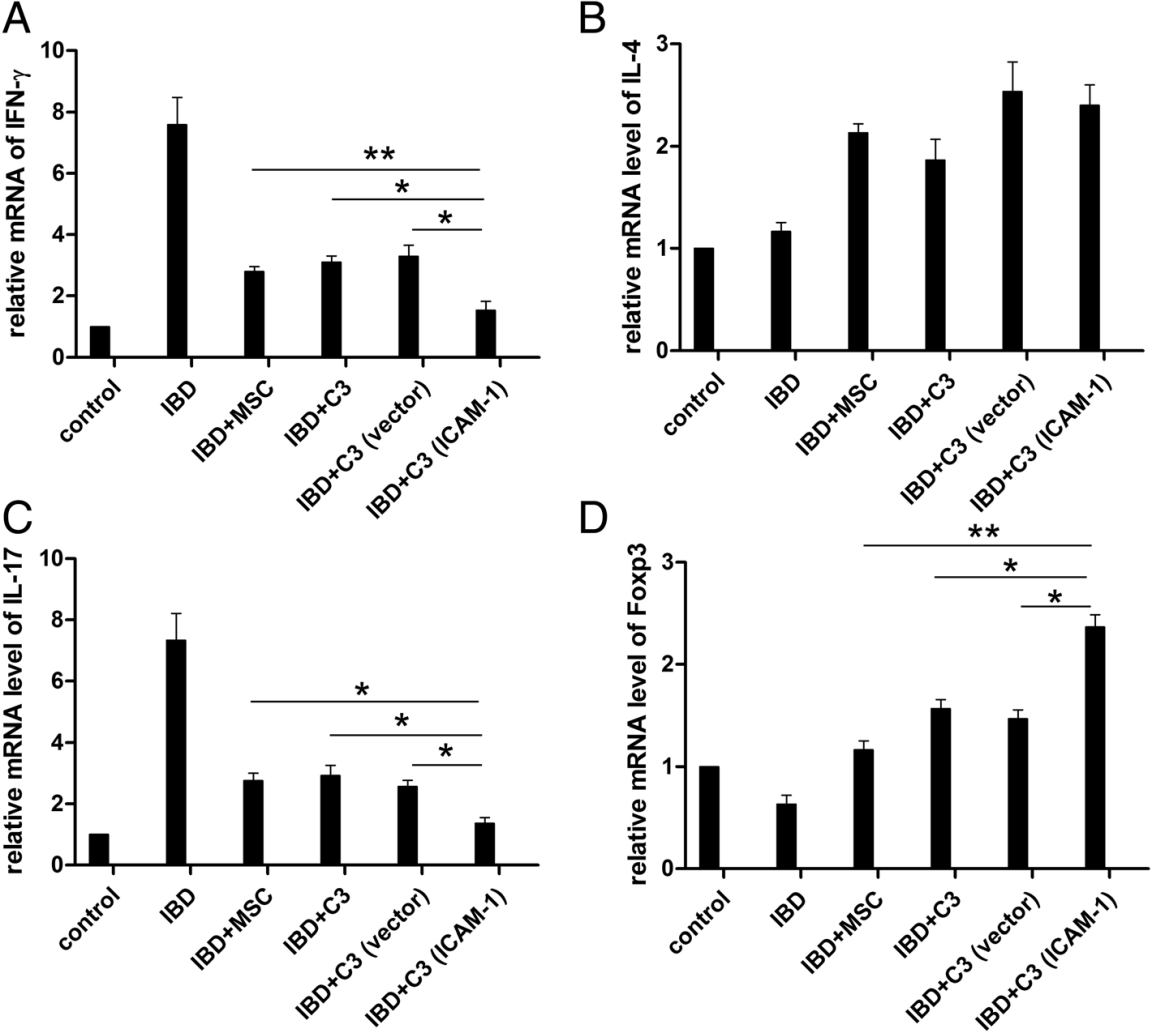
The infusion of ICAM-1-overexpressing MSCs regulates the expression of splenocyte-derived immune factors in vivo*.* The transcriptional levels of IFN-γ and IL-17A are increased in splenocytes of IBD mice, and the gene expression is significantly decreased after infusion of ICAM-1-overexpressing MSC (**a**–**d**). (*, *P* < 0.05, **, *P* < 0.01)

In addition to modulating T cell subpopulations in the spleen, MSC infusion influenced the expression of splenocyte-derived immune factors. After DSS induction, the mRNA expression levels of IFN-γ and IL-17A were significantly increased and their expression declined after MSC infusion. The changes in IFN-γ and IL-17A expression were more pronounced in mice that received C3-ICAM-1 cells (Fig. 7). Consistent with the changes in Th2 subpopulation, no significant differences in IL-4 expression were observed in mice of the C3-vector and C3-ICAM-1 groups. Foxp3 expression is required for Treg production; Tregs protect tissue from hyperactive inflammatory response. As shown in Fig. 7, ICAM-1-overexpressing MSCs dramatically restored the number of Tregs in the spleen of IBD mice compared to that in the case of C3-vector cells. This effect suggests that ICAM-1 has a protective role in MSC-based IBD treatment.

## Discussion

In the current study, we found that systemic infusion of ICAM-1-overexpressing MSCs dramatically attenuated pathological lesions in the mouse model of IBD. Additionally, higher number of ICAM-1-overexpressing MSCs was detected in the colon and spleen of IBD mice. Moreover, the T cell subpopulations in the spleen were remarkably repolarized after MSC infusion.

IBD is an intractable autoimmune disorder that markedly deteriorates quality of life. The etiology and pathogenic mechanisms underlying IBD remain largely elusive; however, increasing evidence indicates that the development of IBD results from an extremely complex interaction between genetics, environment, microbial factors, and autoimmune responses eventually leading to chronic inflammation and tissue destruction in the gastrointestinal tract [21, 22]. The central feature of IBD is defined by inflammation of the gastrointestinal tract, which is associated with unrestrained immune response with increased abnormal T cell activity.

MSCs are characterized by active proliferation, plastic differentiation, strong immunomodulation, low immunogenicity, and abundant trophic factor production; MSCs alleviate inflammation by modulating inflammatory cytokines in inflamed tissues [23, 24]. Additionally, MSCs contribute to the regeneration of injured organs via cellular migration [17, 18]. Hence, MSCs have been tested as efficient treatments for immune-related diseases, including IBD [23, 25]. Numerous studies have shown that MSC administration may be an important treatment option for IBD*.* MSC-based therapy targets multiple pathological processes in IBD by replacing damaged tissues, inhibiting inflammation, and suppressing fibrosis [25–27].

However, MSCs used for IBD treatment are currently administered through intravenous transplantation resulting in a likelihood of remaining in the blood-rich tissues (liver, lung, and spleen). A number of animal studies reported low levels of MSC recruitment and persistence in vivo [28]. Therefore, methods for enhancing homing to the target organ and anti-inflammatory effects of MSCs are urgently needed to improve their clinical efficacy. Intercellular adhesion molecule-1 (ICAM-1), also known as CD54, participates in signal transmission between the cells, regulates immune response, mediates cell differentiation and development, and is closely related to lymphocyte homing and recycling [9, 10]. Normally, ICAM-1 is not expressed on the surface of MSCs; however, ICAM-1 is upregulated in the inflammatory microenvironment. ICAM-1 enhances the immunosuppressive effect of MSCs and significantly enhances the adhesion of T cells. When ICAM-1 is functionally blocked or knocked out, the immunosuppressive effect of MSC is significantly reversed [9, 10].

MSCs have a stable genetic background and foreign genes are easy to introduce and overexpress in these cells. Therefore, overexpression of ICAM-1 may play a role in promoting the homing of MSCs to the target organs thus enhancing MSC presence in the injured tissues in IBD, such as an inflamed colon. However, the exact effects and underlying mechanisms require additional experimental confirmation. Fortunately, we directly observed that a higher number of C3-ICAM-1 cells remained in the colon and spleen of IBD mice and the cells were significantly more abundant than the cells administered in other groups. Functionally, ICAM-1-overexpressing MSCs improve the general condition of IBD mice, promote weight recovery, improve survival rate, and reduce intestinal tissue damage.

Increasing evidence has demonstrated that the balanced regulation between proinflammatory immune cells/cytokines and anti-inflammatory immune cells/cytokines plays an important role in the development of IBD [29, 30]. T cells are the key mediators of the adaptive immune system and are ubiquitous in animal and human tissues. Once activated, T cells can differentiate into Th1, Th2, Th9, Th17, or Tregs according to the intensity of stimulation and the cytokine microenvironment. Hence, T cells modulate various autoimmune diseases and protect organisms from infections and malignancies [31]. Moreover, it has been demonstrated that MSCs tightly interact with T cells [32–36].

Spleen is one of the main peripheral immune organs and contains a high number of T cells; hence, we explored the alterations of T cell subpopulations and splenocyte-derived immune factors in IBD mice after MSC infusion. One of the important findings of our study is that ICAM-1-overexpressing MSC transplantation significantly reduces the abundance of Th1, Th17, and splenic Treg cells in IBD mice. Additionally, transplantation inhibits the expression of INF-γ and IL-17A and promotes the expression of Foxp3. Therefore, we established a novel connection between ICAM-1 and MSC-mediated therapy in IBD. However, ICAM-1-overexpressing MSCs did not increase the number of Th2 cells and the expression of IL-4 compared with an increase detected in the case of C3-vector cells in the present study. We speculate that this difference may be related to the roles of Th2 cells and IL-4 in the pathogenesis of IBD and this mechanism requires additional investigation.

It should be noted that the route of MSC administration and the source and type of MSCs may influence the therapeutic effects and should be clarified in the future studies. Gonçalves et al. compared the therapeutic effects of different routes of administration of MSCs in the treatment of experimental colitis [37]. The data demonstrated that intravenous administration of mouse adipose tissue-derived MSCs was more effective than that in the case of intraperitoneal treatment in reducing the clinical and histopathological severity of colitis. Further studies revealed that the downregulation of proinflammatory cytokines (IL-6 and TNF-α) and the upregulation of anti-inflammatory cytokines (IL-10 and IL-4) may contribute to the therapeutic effects of MSCs [37]. In our study, the MSCs were intravenously administered to IBD mice and the data indicate that overexpression of ICAM-1 in MSCs remarkably augmented the beneficial effects of MSC therapy by overcoming low homing efficiency of MSCs suggesting that improving the homing activity of MSCs is an effective therapeutic strategy in inflammatory colitis.

Additionally, Legaki et al. reported the therapeutic potential of secreted molecules derived from human amniotic fluid mesenchymal stem/stromal cells (SS-AF-MSCs) in a mouse model of colitis [38]. The authors found that administration of conditioned media (CM) derived from SS-AF-MSCs can reduce the severity of colitis in mice. Mechanistic analysis showed that anti-inflammatory cytokine levels, such as TGF-β1 and IL-10, were significantly increased while proinflammatory cytokine levels, including TNF and IL-1, were remarkably decreased in IBD mice after CM infusion [38]. These data suggest that SS-AF-MSCs and their CM have a potential use in IBD therapy. Another study demonstrated that intraperitoneal administration of the extracts of MSCs (MSC-Ex) instead of MSCs yielded improved therapeutic effects in experimental colitis. The results showed that MSC-Ex blocked the expression of inflammatory cytokines and shifted the macrophage functional phenotype from M1 to M2 [39]. Notably, the treatment with MSC-Ex was more potent than that with MSC in reducing DAI, the histological score, and nitrite levels [39]. We did not administer MSCs or their derivatives in IBD mice intraperitoneally in the current study; however, we believe that interesting findings mentioned above may be helpful in reinforcing our findings in future studies.

## Conclusion

MSC-based treatment of IBD has good application potential; however, numerous clinical and basic research problems remain to be solved. Our results indicate that ICAM-1-overexpressing MSCs have higher migration to inflamed intestinal tissue and the spleen, which enhances their therapeutic effects. However, whether MSCs can survive for an extended duration in the recipient tissues and their final fate in vivo after implantation require further study. These results may help in the development of cell-based therapies for the treatment of IBD and the expansion of MSC treatment to a wider range of diseases.

## Additional files

Additional file 1: **Figure S1.** High level of ICAM-1 is expression on C3-ICAM-1 cells. ICAM-1 was expressed at high level in the gene-modified C3 cells. The results of flow cytometry show that more that 70% cells are ICAM-1-positive in the group of ICAM-1-overexpressing MSCs. (TIF 921 kb)

Additional file 2: **Figure S2.** ICAM-1-overexpressing MSCs suppress the expression of TNF-α in the colon of IBD mice. Significant reduction in inflammatory cytokine TNF-α is demonstrated by immunohistochemical staining in the colon of IBD mice after transplantation of ICAM-1-overexpressing MSCs (Figure S2A and S2B). Bars in Figure S2A represent 500 μm. (*, P<0.05, **, P<0.01). (TIF 3372 kb)

Additional file 3: **Table S1.** Primer sequences. (DOC 31 kb)

## Data Availability

The datasets used and/or analyzed during the current study are available from the corresponding author on reasonable request.

## Abbreviations

CAMs: Cell adhesion molecules
CM-DIL: 1,1′-Dioctadecyl-3,3,3′,3′-tetramethylindocarbocyanine perchlorate
DAPI: 4′,6-Diamidino-2-phenylindole
Foxp3: Forkhead box P3
HE: Hematoxylin and eosin
IBD: Inflammatory bowel disease
ICAM-1: Intercellular adhesion molecule-1
IFN-γ: Interferon-γ
IL-17A: Interleukin 17A
MHC: Major histocompatibility complex
MSCs: Mesenchymal stem cells
PCR: Polymerase chain reaction
PDGF: Platelet-derived growth factor
PE: Phycoerythrin
PMA: Phorbol 12-myristate 13-acetate
TNF-α: Tumor necrosis factor α
Treg: Regulatory T cells

## References

[1] Corridoni D, Arseneau KO, Cominelli F (2014). Inflammatory bowel disease. Immunol Lett.

[2] Ananthakrishnan AN, Bernstein CN, Iliopoulos D, Macpherson A, Neurath MF, Ali RAR, Vavricka SR, Fiocchi C (2018). Environmental triggers in IBD: a review of progress and evidence. Nat Rev Gastroenterol Hepatol..

[3] Neurath MF (2017). Current and emerging therapeutic targets for IBD. Nat Rev Gastroenterol Hepatol.

[4] Menon T, Afzali A. Inflammatory bowel disease: a practical path to transitioning from pediatric to adult care. Am J Gastroenterol. 2019. 10.14309/ajg.0000000000000222 [Epub ahead of print]. 10.14309/ajg.0000000000000222 30985298

[5] Markovic BS, Kanjevac T, Harrell CR, Gazdic M, Fellabaum C, Arsenijevic N, Volarevic V (2018). Molecular and cellular mechanisms involved in mesenchymal stem cell-based therapy of inflammatory bowel diseases. Stem Cell Rev.

[6] Soontararak S, Chow L, Johnson V, Coy J, Wheat W, Regan D, Dow S (2018). Mesenchymal stem cells (MSC) derived from induced pluripotent stem cells (iPSC) equivalent to adipose-derived msc in promoting intestinal healing and microbiome normalization in mouse inflammatory bowel disease model. Stem Cells Transl Med.

[7] Nitzsche F, Müller C, Lukomska B, Jolkkonen J, Deten A, Boltze J (2017). Concise review: MSC adhesion cascade-insights into homing and transendothelial migration. Stem Cells.

[8] Zhang X, Chen J, Xue M, Tang Y, Xu J, Liu L, Huang Y, Yang Y, Qiu H, Guo F (2019). Overexpressing p130/E2F4 in mesenchymal stem cells facilitates the repair of injured alveolar epithelial cells in LPS-induced ARDS mice. Stem Cell Res Ther.

[9] Ren G, Zhao X, Zhang L, Zhang J, L’Huillier A, Ling W, Roberts AI, Le AD, Shi S, Shao C, Shi Y (2010). Inflammatory cytokine-induced intercellular adhesion molecule-1 and vascular cell adhesion molecule-1 in mesenchymal stem cells are critical for immunosuppression. J Immunol.

[10] Rubtsov Y, Goryunov К, Romanov А, Suzdaltseva Y, Sharonov G, Tkachuk V (2017). Molecular mechanisms of immunomodulation properties of mesenchymal stromal cells: a new insight into the role of ICAM-1. Stem Cells Int.

[11] Xu FF, Zhu H, Li XM, Yang F, Chen JD, Tang B, Sun HG, Chu YN, Zheng RX, Liu YL, Wang LS, Zhang Y (2014). Intercellular adhesion molecule-1 inhibits osteogenic differentiation of mesenchymal stem cells and impairs bio-scaffold-mediated bone regeneration in vivo. Tissue Eng Part A.

[12] Wang YG, Zhao Y, Li XM, Tang B, Chu YN, Liu YL, Zhu H, Zhang Y (2014). Effect of intercellular adhesion molecule-1 on the migration in vitro of murine mesenchymal stem cells and its related mechanism. Zhongguo Shi Yan Xue Ye Xue Za Zhi.

[13] Zhu H, Guo ZK, Jiang XX, Li H, Wang XY, Yao HY, Zhang Y, Mao N (2010). A protocol for isolation and culture of mesenchymal stem cells from mouse compact bone. Nat Protoc.

[14] Li X, Zhang ZC, Zu ZF, Mao XY, Zhu H, Ning SB (2015). The influence of different concentrations of DSS on the establishment of IBD model and the expression of colitis related immune factor. Acta Lab Anim Sci Sin.

[15] Scheiffele F, Fuss IJ (2002). Induction of TNBS colitis in mice. Curr Protoc Immunol.

[16] Baumgart DC, Carding SR (2007). Inflammatory bowel disease: cause and immunobiology. Lancet..

[17] Li H, Guo Z, Jiang X, Zhu H, Li X, Mao N (2008). Mesenchymal stem cells alter migratory property of T and dendritic cells to delay the development of murine lethal acute graft-versus-host disease. Stem Cells.

[18] Auletta JJ, Deans RJ, Bartholomew AM (2012). Emerging roles for multipotent, bone marrow-derived stromal cells in host defense. Blood..

[19] Chen ML, Sundrud MS (2016). Cytokine networks and T-cell subsets in inflammatory bowel diseases. Inflamm Bowel Dis.

[20] Smids C, Horjus Talabur Horje CS, Drylewicz J, Roosenboom B, MJM G, van Koolwijk E, van Lochem EG, Wahab PJ (2018). Intestinal T cell profiling in inflammatory bowel disease: linking T cell subsets to disease activity and disease course. J Crohns Colitis.

[21] Kaplan GG (2015). The global burden of IBD: from 2015 to 2025. Nat Rev Gastroenterol Hepatol..

[22] Kaplan GG, Ng SC (2017). Understanding and preventing the global increase of inflammatory bowel disease. Gastroenterology..

[23] Adak S, Mukherjee S, Sen D (2017). Mesenchymal Stem Cell as a Potential Therapeutic for Inflammatory Bowel Disease- Myth or Reality?. Curr Stem Cell Res Ther.

[24] Zhang H, Li ZL, Yang F, Zhang Q, Su XZ, Li J, Zhang N, Liu CH, Mao N, Zhu H (2018). Radial shockwave treatment promotes human mesenchymal stem cell self-renewal and enhances cartilage healing. Stem Cell Res Ther.

[25] Robinson AM, Rahman AA, Miller S, Stavely R, Sakkal S, Nurgali K (2017). The neuroprotective effects of human bone marrow mesenchymal stem cells are dose-dependent in TNBS colitis. Stem Cell Res Ther.

[26] Abdel Salam AG, Ata HM, Salman TM, Rashed LA, Sabry D, Schaalan MF (2014). Potential therapeutic utility of mesenchymal stem cells in inflammatory bowel disease in mice. Int Immunopharmacol.

[27] Lightner AL (2019). Stem cell therapies for inflammatory bowel disease. Curr Gastroenterol Rep.

[28] Kang SK, Shin IS, Ko MS, Jo JY, Ra JC (2012). Journey of mesenchymal stem cells for homing: strategies to enhance efficacy and safety of stem cell therapy. Stem Cells Int.

[29] Singh UP, Singh NP, Singh B, Mishra MK, Nagarkatti M, Nagarkatti PS, Singh SR (2010). Stem cells as potential therapeutic targets for inflammatory bowel disease. Front Biosci (Schol Ed).

[30] Benchimol EI, Mack DR, Nguyen GC, Snapper SB, Li W, Mojaverian N, Quach P, Muise AM (2014). Incidence, outcomes, and health services burden of very early onset inflammatory bowel disease. Gastroenterology..

[31] Pribila JT, Quale AC, Mueller KL, Shimizu Y (2004). Integrins and T cell-mediated immunity. Annu Rev Immunol.

[32] Carrión F, Nova E, Luz P, Apablaza F, Figueroa F (2011). Opposing effect of mesenchymal stem cells on Th1 and Th17 cell polarization according to the state of CD4+ T cell activation. Immunol Lett.

[33] Duffy MM, Ritter T, Ceredig R, Griffin MD (2011). Mesenchymal stem cell effects on T-cell effector pathways. Stem Cell Res Ther.

[34] Luz-Crawford P, Kurte M, Bravo-Alegría J, Contreras R, Nova-Lamperti E, Tejedor G, Noël D, Jorgensen C, Figueroa F, Djouad F, Carrión F (2013). Mesenchymal stem cells generate a CD4+CD25+Foxp3+ regulatory T cell population during the differentiation process of Th1 and Th17 cells. Stem Cell Res Ther.

[35] Chen M, Su W, Lin X, Guo Z, Wang J, Zhang Q, Brand D, Ryffel B, Huang J, Liu Z, He X, Le AD, Zheng SG (2013). Adoptive transfer of human gingiva-derived mesenchymal stem cells ameliorates collagen-induced arthritis via suppression of Th1 and Th17 cells and enhancement of regulatory T cell differentiation. Arthritis Rheum.

[36] Vega-Letter AM, Kurte M, Fernández-O'Ryan C, Gauthier-Abeliuk M, Fuenzalida P, Moya-Uribe I, Altamirano C, Figueroa F, Irarrázabal C, Carrión F (2016). Differential TLR activation of murine mesenchymal stem cells generates distinct immunomodulatory effects in EAE. Stem Cell Res Ther.

[37] Gonçalves Fda C, Schneider N, Pinto FO, Meyer FS, Visioli F, Pfaffenseller B, Lopez PL, Passos EP, Cirne-Lima EO, Meurer L, Paz AH (2014). Intravenous vs intraperitoneal mesenchymal stem cells administration: what is the best rout for treating experimental colitis?. World J Gastroenterol.

[38] Legaki E, Roubelakis MG, Theodoropoulos GE, Lazaris A, Kollia A, Karamanolis G, Marinos E, Gazouli M (2016). Therapeutic potential of secreted molecules derived from human amniotic fluid mesenchymal stem/stroma cells in a mice model of colitis. Stem Cell Rev.

[39] Song JY, Kang HJ, Hong JS, Kim CJ, Shim JY, Lee CW, Choi J (2017). Umbilical cord-derived mesenchymal stem cell extracts reduce colitis in mice by re-polarizing intestinal macrophages. Sci Rep.

