# Supplementary figures and images for: Intercellular adhesion molecule-1 enhances the therapeutic effects of MSCs in a dextran sulfate sodium-induced colitis models by promoting MSCs homing to murine colons and spleens
Source: Stem Cell Res Ther. 2019 Aug 23;10:267. doi: 10.1186/s13287-019-1384-9 (PMC6708236; doi:10.1186/s13287-019-1384-9)

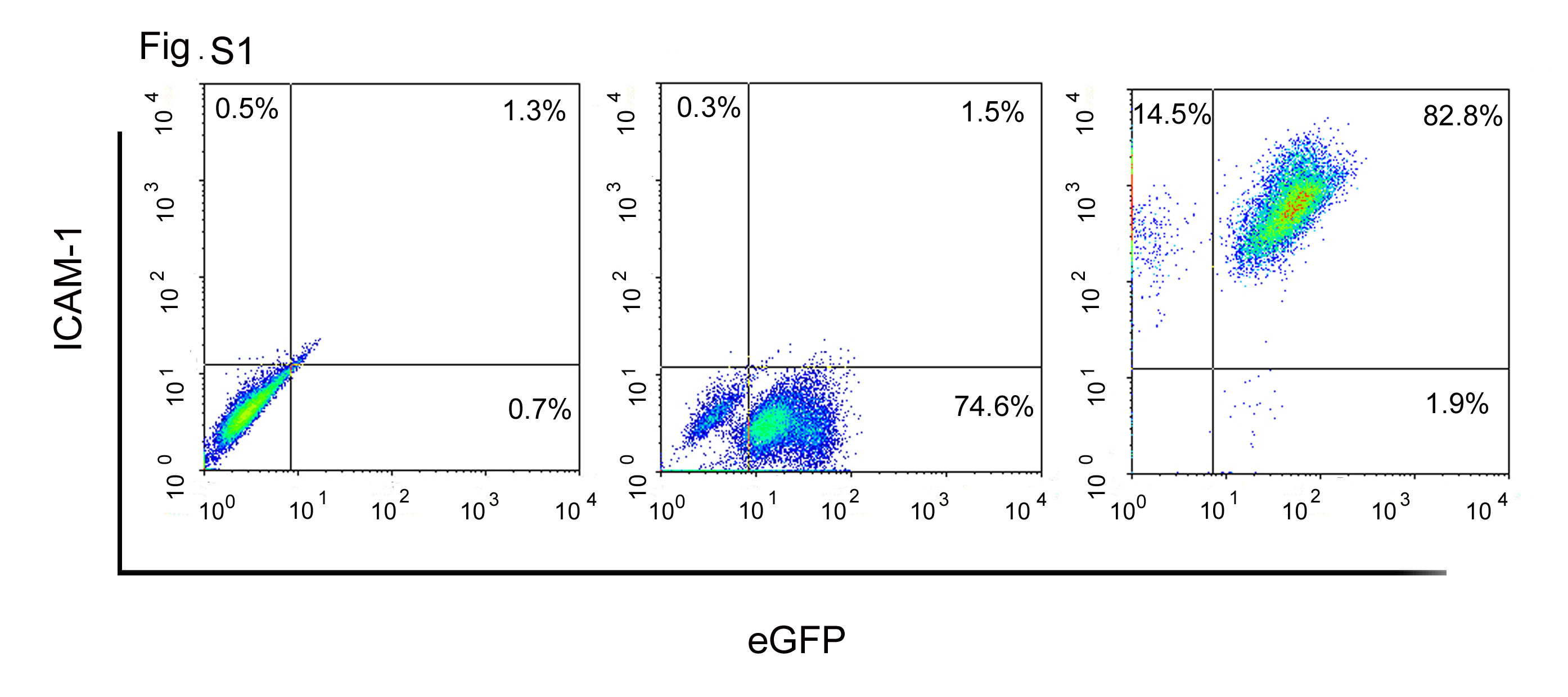

Supplement: Supplementary file 1 — Figure S1. High level of ICAM-1 is expression on C3-ICAM-1 cells. ICAM-1 was expressed at high level in the gene-modified C3 cells. The results of flow cytometry show that more that 70% cells are ICAM-1-positive in the group of ICAM-1-overexpressing MSCs. (TIF 921 kb) [file 13287_2019_1384_MOESM1_ESM.tif]

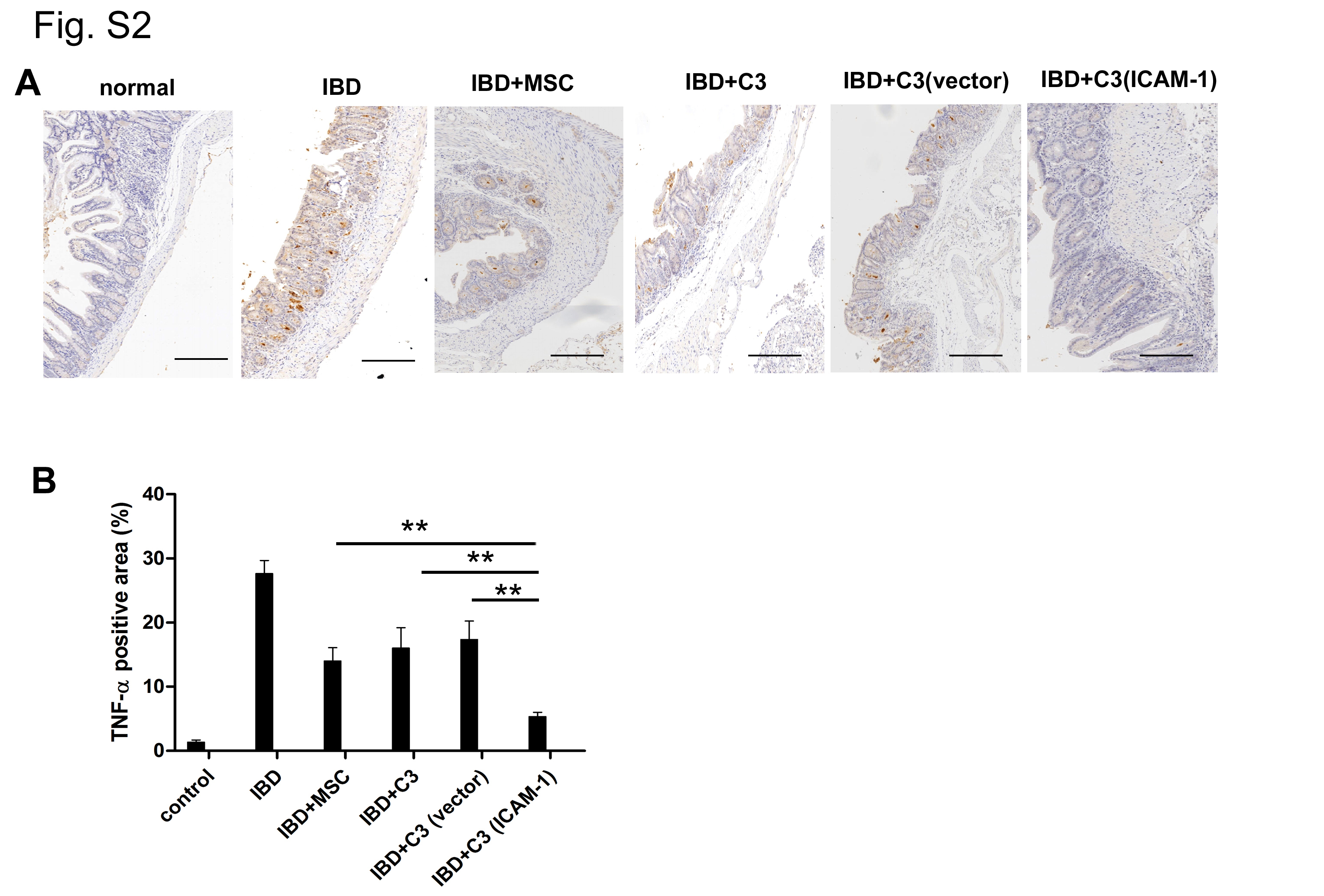

Supplement: Supplementary file 2 — Figure S2. ICAM-1-overexpressing MSCs suppress the expression of TNF-α in the colon of IBD mice. Significant reduction in inflammatory cytokine TNF-α is demonstrated by immunohistochemical staining in the colon of IBD mice after transplantation of ICAM-1-overexpressing MSCs (Figure S2A and S2B). Bars in Figure S2A represent 500 μm. (*, P<0.05, **, P<0.01). (TIF 3372 kb) [file 13287_2019_1384_MOESM2_ESM.tif]
