# Supplementary material for: Intercellular adhesion molecule-1 enhances the therapeutic effects of MSCs in a dextran sulfate sodium-induced colitis models by promoting MSCs homing to murine colons and spleens
Source: Stem Cell Res Ther. 2019 Aug 23;10:267. doi: 10.1186/s13287-019-1384-9 (PMC6708236; doi:10.1186/s13287-019-1384-9)
Supplement: Supplementary file 3 — Table S1. Primer sequences. (DOC 31 kb) [file 13287_2019_1384_MOESM3_ESM.doc]

| genes | primer sequences | Annealing temperature |
| --- | --- | --- |
| HPRT  ICAM-1  IL-4  IFN-γ  IL-17A  Foxp3 | forward, 5′- AGTCAAGGGCATATCCAACAACAA -3′  reverse, 5′- GCTGGTGAAAAGGACCTCTCG -3′  forward, 5′-GCTTCACACTTCACAGTTACTT-3′  reverse, 5′-AGAGGACCTTAACAGTCTACAAC-3′  forward, 5′- ACTCCATGCTTGAAGAAGAACTC -3′  reverse, 5′- ATGATGCTCTTTAGGCTTTCCA -3′  forward, 5′- TTACTACCTTCTTCAGCAACAGCAA -3′  reverse, 5′- CTGGTGGACCACTCGGATGA -3′  forward, 5′- TCCAGAAGGCCCTCAGACTA -3′  reverse, 5′- AGCATCTTCTCGACCCTGAA -3′  forward, 5′- AGAGCCCTCACAACCAGCTA -3′  reverse, 5′- CCAGATGTTGTGGGTGAGTG -3′ | 60 ℃ |

**Table S1: Primer sequences**
